# Planning for Implementation Success Using RE-AIM and CFIR Frameworks: A Qualitative Study

**DOI:** 10.3389/fpubh.2020.00059

**Published:** 2020-03-03

**Authors:** Diane K. King, Jo Ann Shoup, Marsha A. Raebel, Courtney B. Anderson, Nicole M. Wagner, Debra P. Ritzwoller, Bruce G. Bender

**Affiliations:** ^1^Center for Behavioral Health Research and Services, University of Alaska Anchorage, Anchorage, AK, United States; ^2^Kaiser Permanente Colorado, Institute for Health Research, Denver, CO, United States; ^3^Department of Pediatrics, National Jewish Health, Denver, CO, United States

**Keywords:** adoption, implementation, maintenance, sustainability, dissemination, frameworks

## Abstract

**Background:** RE-AIM (Reach, Effectiveness, Adoption, Implementation, Maintenance) and CFIR (Consolidated Framework for Implementation Research) dissemination and implementation frameworks define theory-based domains associated with the adoption, implementation and maintenance of evidence-based interventions. Used together, the two frameworks identify metrics for evaluating implementation success, i.e., high reach and effectiveness resulting in sustained practice change (RE-AIM), and modifiable factors that explain and enhance implementation outcomes (CFIR). We applied both frameworks to study the implementation planning process for a technology-delivered asthma care intervention called Breathewell within an integrated care organization. The goal of the Breathewell intervention is to increase the efficiency of delivering resource-intensive asthma care services.

**Methods:** We reviewed historical documents (i.e., meeting agendas; minutes) from 14 months of planning to evaluate alignment of implementation team priorities with RE-AIM domains. Key content was extracted and analyzed on topics, frequency and amount of discussion within each RE-AIM domain. Implementation team members were interviewed using questions adapted from the *CFIR Interview Guide Tool* to focus their reflection on the process and contextual factors considered during pre-implementation planning. Documents and transcripts were initially coded using RE-AIM domain definitions, and recoded using CFIR constructs, with intent to help explain how team decisions and actions can contribute to adoption, implementation and maintenance outcomes.

**Results:** Qualitative analysis of team documents and interviews demonstrated strong alignment with the RE-AIM domains: Reach, Effectiveness, and Implementation; and with the CFIR constructs: *formal inclusion* of provider and staff stakeholders in implementation planning, *compatibility* of the intervention with workflows and systems, and alignment of the intervention with organizational *culture*. Focus on these factors likely contributed to RE-AIM outcomes of high implementation fidelity. However, team members expressed low confidence that Breathewell would be adopted and maintained post-trial. A potential explanation was weak alignment with several CFIR constructs, including *tension for change, relative priority, and leadership engagement* that contribute to organizational receptivity and motivation to sustain change.

**Conclusions:** While RE-AIM provides a practical framework for planning and evaluating practice change interventions to assure their external validity, CFIR explains *why* implementation succeeded or failed, and when used proactively, identifies relevant modifiable factors that can promote or undermine adoption, implementation, and maintenance.

## Introduction

Dissemination and implementation (D&I) research has demonstrated that evidence of effectiveness is insufficient to promote adoption of evidence-based interventions if fit and feasibility have not been addressed (1, 2). A growing body of research has also found that even feasible interventions may not be fully adopted or sustained if organizational demands related to market forces (e.g., competitive, consumer, capacity, or regulatory) or other strategic imperatives (e.g., patient wants and needs) are not considered (3, 4).

D&I frameworks, such as RE-AIM (Reach, Effectiveness, Adoption, Implementation, Maintenance) (5, 6), can be used during implementation planning (7) to guide selection, adaptation, and evaluation of interventions on key indicators associated with successful implementation of evidence-based interventions. By defining whose health or health behavior will benefit from the intervention (*Reach*), identifying which components of the intervention are considered the “active ingredients” necessary for the desired impact (*Effectiveness*), describing relevant characteristics of the delivery setting, and those involved in delivering the intervention (*Adoption*); evaluating the extent that the active ingredients are delivered with fidelity to the established protocols (*Implementation*), and describing facilitators and barriers that may influence organizational decisions to sustain the intervention after the study is completed (*Maintenance*), RE-AIM provides practical information that can improve translation of evidence-based interventions into practice and their public health impact (8). The framework's emphasis on balancing rigor with relevance is clearly important to adoption, implementation and maintenance (9). Implementation success (i.e., post-trial sustainment of an intervention, with protocols and infrastructure in place to assure continued fidelity) can depend on the extent that an organization has internal capacity (10) and is willing to accommodate the intervention by modifying setting systems, protocols, and/or roles (11); and the extent that researchers are willing to adapt the intervention, so that it fits and is feasible to maintain long-term (3).

However, RE-AIM does not explain the conditions that influence implementation success (12). Other frameworks, such as the Chronic Care Model (CCM) (13) and the Practical, Robust Implementation and Sustainability Model (PRISM) (14), include constructs from improvement science important to intervention design and acceptance, such as external and internal support for the intervention, internal preparedness/readiness, compatibility with internal systems, and observed effectiveness of the intervention. However, they lack clear definitions, guidance or measures to assist planning teams in understanding or improving results (15). Use of qualitative methods, such as asking stakeholders and observing processes to identify barriers to implementation, have been recommended to further our understanding of why implementers got the results they did (12, 16). While anticipating barriers is important, understanding individual, situational and structural influences on outcome expectations, behavior and decision-making can identify specific mechanisms that could be assessed and addressed during implementation planning (4, 15). In addition to improvement science, marketplace principles that include understanding customers (i.e., payors) and competition (i.e., other priorities, programs) for the intervention, can be useful to improving success (or understanding failure) (17). Lessons from marketing science describe how researchers have a tendency to rely on “push,” defined as systematic efforts to convince potential adopters of the value of our interventions (i.e., dissemination), vs. “pull,” defined as pre-existing preferences, needs, or demands that intrinsically motivate potential adopters to change (i.e., diffusion) (3). Improving receptivity to adopting interventions may require using push techniques to elicit pull, by tailoring dissemination to address the wants, needs, and concerns of decision-makers within the organization (18).

**Figure 1:**
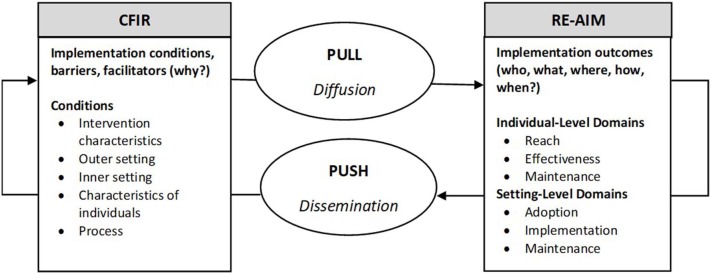
Implementation planning conceptual framework: Using RE-AIM and CFIR to plan for successful implementation.

The Consolidated Framework for Implementation Research (CFIR) is a comprehensive framework composed of constructs associated with effective implementation (19). CFIR's 39 constructs are organized into five domains: *Intervention Characteristic*; *Outer Setting*; *Inner Setting*; *Characteristics of Individuals*; and *Process* (20). Like CCM and PRISM, CFIR draws on theories of behavior change, improvement science, and Diffusion Theory, but also provides a taxonomy with definitions, codebook, and interview questions, to facilitate its usefulness as an explanatory model (21). Understanding which constructs, or sets of constructs promote or inhibit adoption, implementation, and maintenance, can inform development during planning of tailored and testable implementation strategies (22) to balance internal and external validity (4), as well as push and pull (3). In other words, examining the presence or absence of CFIR constructs can explain “why” implementation was or was not successful, while RE-AIM describes outcomes in terms of “who, what, where, how, and when” (12) (see Figure 1).

Used together, RE-AIM and CFIR could enhance the effectiveness of implementation planning by elucidating relationships between factors emphasized (or missed), which potentially could promote implementation fidelity and adoption, and thus lead to optimal post-trial maintenance outcomes. RE-AIM and CFIR domains, definitions, constructs and the “who, what, where, how, when, and why” questions for planning teams are summarized in Table 1.

**Table 1:** RE-AIM and CFIR domains, planning questions, and definitions/constructs.

**RE-AIM Framework**	
**Reach**^**a**^	
Planning questions	**Who (which patient) is intended to benefit from the intervention? Who will be exposed to the intervention?**
Definition	The absolute number, proportion, and representativeness (whether participants have characteristics that reflect the target population's characteristics) of individuals exposed to the intervention; as well as characteristics of those who were eligible but not reached
**Effectiveness**^**a**^	
Planning questions	**What are the most important benefits you hope to achieve? How will we know if the intervention achieved these benefits?**
Definition	The impact of an intervention on important outcomes. This includes potential negative effects, quality of life, and economic outcomes
**Adoption**	
Planning questions	**Where is the intervention being delivered? How do we develop institutional support to deliver it?**
Definition	The absolute number, proportion, and representativeness of settings and staff who are willing to initiate a program or approve a policy
**Implementation**	
Planning questions	**How do we assure the intervention is delivered properly and consistently? How do we adapt it to make sure it fits and is feasible?**
Definition	To what extent is the intervention delivered as designed; includes how closely and consistently staff members follow established protocols, as well as the time and cost of the program
**Maintenance**	
Planning questions	**When will the intervention become operational? How do we assure the intervention continues to be effective and delivered as designed, over time?**
Definition	At the setting level, the extent to which a program or policy becomes part of the routine organizational practices and policies
**Consolidated framework for implementation research**	
**Intervention characteristics**	
Planning questions	**Is this intervention superior to the status quo? Can we adapt it so that it will work here?**
Constructs	Intervention source; evidence strength; relative advantage; adaptability; trialability; complexity; design quality; and cost
**Outer setting**^**b**^	
Planning questions	**Why is it important for our institution to do this intervention now? Does it address a gap in patient care? Are there regulatory or competitive reasons?**
Constructs	Patient needs; organizational networks; peer or competitive pressure; policies, regulations and incentives
**Inner setting**	
Planning questions	**Will the intervention fit within our system? Is it feasible to do this now?**
Constructs	Structural characteristics; networks and communication; culture; implementation climate (tension for change; compatibility; relative priority; incentives and rewards; goals and feedback; learning climate); readiness for implementation (leadership engagement; available resources; intervention knowledge and access)
**Characteristics of individuals**	
Planning questions	**Do our providers and staff have the skill and will to deliver it?**
Constructs	Knowledge, attitudes and beliefs about the intervention; self-efficacy to deliver the intervention; individual stage of change; identification with the organization; personal attributes and values
**Process**	
Planning questions	**Whose work is affected by the intervention? Whose buy-in, input and expertise is needed? Who can commit the resources required to implement and sustain the intervention?**
Constructs	Planning; Engaging (opinion leaders, formally appointed stakeholders, champions, external change agents); Executing; Reflecting

a These RE-AIM domains were not used in our assessment as these domains are at the individual level.

b These CFIR domains were not used in our assessment as these domains relate to external factors to implementation; our intervention was delivered via technology, so characteristics of individuals were not as significant.

*RE-AIM planning questions were adapted from a recent publication on pragmatic applications of the framework (7); CFIR planning questions were conceived by the authors*.

The objective of this paper is to describe our complementary use of the RE-AIM evaluation framework and the CFIR explanatory framework to go beyond listing barriers; to identify potentially testable mechanisms that influence implementation success, and in turn contribute to the forward progression of Implementation science. Using a recent technology-based asthma intervention, the Breathewell study, as the example, we: (1) identify the presence or absence of variables that contribute to implementation success; (2) develop potential implementation strategies that could improve comprehensiveness of the implementation planning process; and (3) recommend areas for future research.

## Materials and Methods

### Context

#### Setting Characteristics and Breathewell Study Description

The setting for the Breathewell study was Kaiser Permanente Colorado (KPCO), an integrated healthcare organization serving ~600,000 members in the Denver-Boulder area. The Breathewell study is a pragmatic randomized controlled trial to experimentally test a technology-enabled outreach intervention targeted to patients diagnosed with asthma who are potentially overusing inhaled beta-agonists (asthma reliever medication). Potential overuse is identified when (1) patients request a refill of their inhaled beta-agonist (asthma reliever) medication more frequently than every 60 days; or (2) request a refill of a beta-agonist without having filled an asthma controller medication (such as an inhaled corticosteroid) within the last 4 months. The technology-based intervention used KPCO's interactive voice response (IVR) system and interfaced between the electronic health record (EHR), patients, and providers (nurses and pharmacists). We conducted our planning for implementing the beta-agonist refill intervention from November 2015 through January 2017, which is the focus of this study. Participants in the trial were Kaiser Permanente Colorado current members, 18 years and older, with a diagnosis of asthma at the time of randomization. Enrollment occurred from February 2017 to February 2018. Participants were randomized to 1 of 3 groups: Text/Phone call intervention, Email, or Usual Care. Participants were followed for 6–18 months, depending on enrollment date. The study was approved by the Institutional Review Boards of National Jewish Health and Kaiser Permanente Colorado. Details of the study design are described elsewhere (23, 24).

#### Reasons for Implementing the Practice Change-Increase Efficiency of Asthma Care

In usual asthma care at KPCO, a group of nurses known as asthma care managers (ACMs) followed-up with patients identified as having too frequent refills of their asthma reliever medication because frequent refills can be an indicator of poor asthma control. The ACMs followed a standard clinical protocol that included time consuming review of the patient's health record along with phone, EHR email portal, or mail contact to the patient to assess patient symptoms and prevent exacerbation. The ACMs indicated to the Breathewell study team that many patients they contacted regarding what appeared to be asthma reliever medication overuse were in fact not overusing the medication, but rather had situations such as requesting an extra asthma reliever inhaler to keep in their gym bag or refilling the medication early due to travel, etc. As a result, the ACMs believed they spent a great deal of time reviewing records and contacting patients who did not have poor asthma control and did not need the expertise of the ACM. The technology-enabled Breathewell study outreach was designed to determine whether the patient currently had symptoms to guide ACM contact.

#### Implementation Team Composition

The 13-member multi-disciplinary planning and implementation team consisted primarily (but not exclusively) of researchers and healthcare professionals from KPCO. The make-up of the implementation team included physician, psychologist, and PharmD co-investigators, an ACM, two biostatisticians, a data manager, a data analyst/informatics specialist, a behavioral scientist, an economist, two project managers, and a research assistant. While patients with asthma did not participate as implementation team members, patients did review and edit the content and wording of the intervention messages prior to their use in study outreach.

### Approach

We used a mix of prospective and retrospective data, and qualitatively analyzed documents and individual interview transcripts, to describe and evaluate the priorities, challenges, and decisions made by the implementation team during the 14-month planning period. First, we compiled all meeting agendas and minutes, then analyzed them by coding for RE-AIM domain alignment. Second, we adapted a subset of CFIR interview questions to further our understanding of setting-level constructs important to planning for implementation of a technology-based intervention designed to improve efficiency of service delivery. Third, we interviewed implementation planning team members individually in a private office or conference room to encourage candor, and coded transcripts by RE-AIM domains and CFIR constructs to help identify what was emphasized (or missed) during planning that likely influenced outcomes for implementation fidelity and potential for post-trial adoption and maintenance. Fourth, we validated these findings with the implementation planning team. Finally, we summarized lessons learned, and formulated a process for developing implementation strategies to improve future implementation planning and implementation success.

### Data

Data included (1) implementation team documents consisting of meeting agendas and detailed bi-monthly team meeting minutes recorded by a research assistant and reviewed after each meeting for accuracy by a project manager and one co-principal investigator; and (2) verbatim transcripts from retrospective interviews with members of the implementation team.

### Analyses

#### Document Review

We used historical document review methods (25) to identify and describe components of the RE-AIM framework that were prioritized during implementation planning. To analyze these documents, we first independently coded the meeting minute content using inductive coding to identify topics and themes discussed during the planning phase. Second, the five RE-AIM domains were applied to the meeting minute content. To compare relative application of RE-AIM domains by the team during planning, from 1 to 10 points were assigned to each domain using a weighting method suggested by Glasgow, et al.: 1–4 = low application, 5–6 = medium application, 7–8 = high application, and 9–10 = very high application of the framework (7).

#### Interviews

After completing the RE-AIM coding of meeting agendas and minutes, we conducted interviews with the planning team, and analyzed them using components of the CFIR framework, organized by the A, I, and M domains of RE-AIM, to explain why planning team priorities impacted implementation success. The interview guide was developed by an external co-investigator removed from day-to-day project operations, and an internal project manager, using the interview guide tool available on the CFIR website (20). Both developers were experienced applying RE-AIM (6, 26) and other implementation frameworks (2, 10). They reviewed the CFIR interview guide tool to identify questions relevant to adopting, implementing and post-trial maintenance of an internally developed, technology-based intervention. Questions were intended to guide reflection about problems and decisions made to maximize intervention fit, feasibility and fidelity at the setting-level, and to describe its potential for sustainability. Because our focus was on the setting-level, we did not include questions that focused on the RE-AIM individual-level domains of Reach or Effectiveness. Also, given that the intervention was developed internally to improve efficiency of service delivery using technology, questions directly related to the CFIR domains of Outer Setting and Characteristics of Individuals were excluded. Twenty-five questions were developed or adapted from the interview guide tool (see Table 2). Eleven of 13 implementation team members were interviewed (the two team members who developed the interview tool were not interviewed). Interviews lasted 45-min on average (range 30–75 min) and were digitally recorded and professionally transcribed.

**Table 2:** Interview guide and a priori RE-AIM and CFIR codes.

**Interview questions organized by RE-AIM domain**	**CFIR domains**	**CFIR constructs**	**CFIR sub-constructs**
**Adoption: characteristics that influence an organization's motivation or capacity to accept or reject an intervention**			
Who was engaged in the decision process to implement an IVR-mediated medication refill service (i.e., BW^a^) at [the organization]? Probe: Was this decision driven by researchers, leadership, or providers?	Intervention characteristics	Intervention source	
What kind of information or evidence did you consider when selecting the BW implementation strategy for your setting?		Evidence strength & quality	
What are the core components of the asthma care intervention (usual care) that contribute to its effectiveness (i.e., need to be present whether human or IVR-delivered)?		Relative advantage	
What costs were considered when deciding to implement BW?		Costs	
To what extent was [the organization's] culture and/or values considered when designing BW. Please describe. In what way is [the organization's] culture different from other settings? In what way is it similar?	Inner setting	Culture	Compatibility
Was there a strong need for this implementation strategy? What was the need driving BW?			Tension for change
To what extent did implementing BW (i.e., IVR-mediated medication refill service) align with organizational goals and priorities?			Relative priority
		Implementation climate	
**Implementation: consistency of delivery as intended**			
When designing BW, did you think about the core components of asthma care that must be retained in both arms, to assure BW arm was NOT inferior to usual care? (i.e., consider the core components of the usual care intervention that made it effective)	Intervention characteristics	Adaptability	
What factors were considered to assure acceptance of BW to Asthma clinicians and care managers (i.e., would minimize resistance/disruption and/or maximize its acceptability and feasibility)?What factors in the use of technology for patient outreach were considered to assure acceptance of BW to patients (i.e., would maximize its acceptability and reach)?		Relative advantage	
Which of these factors do you feel were the most critical to address early on (i.e., would threaten success/derail the project if not addressed?)		Complexity	
When designing BW, to what extent did piloting components factor into the ultimate design.		Trialability	
Are there things that you wish you had piloted with patients or asthma care managers?			
Why did you think the BW implementation strategy would be effective here? Any concerns [regarding using technology for outreach] (e.g., past negative experiences or patient resistance)?	Inner setting	Implementation climate	Compatibility
What kind of approvals were needed? Who was involved?		Readiness for implementation	Leadership engagement
What kinds of infrastructure changes were necessary to accommodate the intervention (e.g., scope of practice; formal policies; information systems or electronic records systems)? Can you describe the process used to make these changes?			Available resources
When designing BW, what key stakeholders did you need to get on board (i.e., whose work or workflows could potentially be impacted by this implementation strategy)? What was your communication or education strategy with these stakeholders?	Process	Engaging	Opinion Leaders Champions
How did you decide who to include on the planning/design team? Were all the appropriate voices at the table from the start?			Formally appointed internal implementation leaders
When planning, did you consider how changes to the process or IVR intervention could be made during the intervention, if needed? Were there elements of the design that could not be altered that were discussed during planning?	Process	Planning	
Describe the process for making decisions about what to track (process and outcomes)? How was the information used?		Reflecting and evaluating	
What process measure(s) was/were most important to monitoring implementation fidelity? Provide an example of how this metric was used to identify issues, problem solve, and/or inform adaptation?			
Has BW been implemented according to plan? To what extent has the plan needed to be modified?		Executing	
**Maintenance: extent that intervention becomes part of an organization's routine practice**			
Whose approval will be needed for maintenance of BW after the study is over (if hypothesized outcomes are demonstrated)? Do these approvers know about the BW study?	Inner setting	Readiness for implementation	Leadership engagement
Do you anticipate any barriers or threats to maintaining BW?			Available resources
Were there factors or costs that weren't considered during implementation, that you wish you had prioritized in hindsight?	Process	Planning	
To what extent will these factors/costs impact BW's adoption or maintenance after the grant?	Intervention characteristics	Relative advantage Cost	

a *BW: Breathewell, a technology-enabled intervention to improve efficiency of asthma medication refills and/or care manager follow-up*.

Two team members independently analyzed all interview transcripts, first applying *a priori* codes that included the setting-level RE-AIM domains (Adoption, Implementation and Maintenance), the selected CFIR domains (Intervention Characteristics, Inner Setting, and Process) and the specific CFIR constructs within those domains that were targeted by the specific interview question (8, 20). Transcripts were then coded a second time, adding any relevant CFIR constructs or subconstructs that emerged from participant responses. After coding three interviews, coders compared coding, discussed discrepancies, and reached consensus on code interpretations. After all interviews were analyzed, coders completed an Excel worksheet that listed each interview question, and its respective RE-AIM and CFIR domain codes, CFIR construct codes, emerging themes, and interviewee quotes that exemplified the assigned codes and themes. Coders then compared their worksheets, discussed any discrepancies and reached final consensus on codes and themes.

Based on the final worksheets, one rater created a matrix that grouped the relevant CFIR constructs under the RE-AIM categories of Adoption, Implementation and/or Maintenance. CFIR constructs listed were those deemed as potentially influencing one or more of the “AIM” domains, hence, some constructs were listed more than once (e.g., the construct of organizational *culture* was listed under Adoption and Implementation). Each rater independently extracted representative quotes that confirmed and/or negated alignment of planning team activities with the CFIR constructs. Each rater then assigned a preliminary rating of weak (one point), moderate (two points), or strong (three points) alignment with the CFIR-constructs based on these quotes and summarized the evidence that supported their ratings. Raters then compared quotes and ratings across the two matrices, discussed any differences, and reached consensus on ratings and evidence. During an implementation team meeting, the combined qualitative matrix of results, ratings and quotes were presented, and the full team reached consensus on data interpretation and major themes.

## Results

### Historical Document Review

Application of the RE-AIM domains during planning varied by domain, with assigned points ranging from 3 to 9, with an average of six, indicating medium overall application of the framework (see Table 3). Ratings indicating very high (nine points) and high (eight points) framework application for Reach and Implementation, reflected the team's chief foci during intervention planning. Parameters for designing the intervention included using technology to enhance usual care by addressing asthma risk factors. Meeting minutes reflected a focus on risk factor data outputs from the EHR and stakeholder input from the ACMs to identify processes and potential opportunities for enhancement. Process metrics to monitor fidelity were established during planning to be used during implementation, e.g., percent of identified patients contacted by the ACM, ACM perceptions of changes to how they allocated their time, and ACM perceptions of benefit of the intervention to patients. In addition, multiple conversations about integrating the technology into the system to resolve technology-related challenges took place throughout the planning process.

**Table 3:** Findings from analysis using RE-AIM^a^ to describe planning team priorities over time.

**Timing**	**Months 1-4**	**Months 5-6**	**Months 7-8**	**Months 9-11**	**Months 12-14**	**Application of RE-AIM domains during planning** **1 (low)−** ** 10 (very high)**
**Themes**	**Preferences and barriers to asthma service delivery**	**Intervention characteristics and implementa-tion strategy development**	**Systems integration, logistics and piloting**	**Reach and intervention logistics**	**System barriers, logistics, monitoring**	
**REACH (R)**						8
	–	Define target population (denominator)	Define patient eligibility and exclusion criteria	Address barriers to reach; opt out options	**——**	
**EFFECTIVENESS (E)**						6
	–	Analyze patient health outcomes, risk factors, and service gaps Use internal data to select intervention	**——**	**——**	–	
**ADOPTION (A)**						4
	Provider-level needs assessed	**——**	**——**	**——**	Get buy-in from ACMs^b^	
**IMPLEMENTATION (I)**						9
	Stakeholder input to describe usual care and potential service- delivery gaps Data availability and quality Potential implementa-tion barriers	Define intervention parameters and analytic plan	Map logistics, information flow, and workflows Develop, test, and refine intervention content	Test and refine logistics, information flow, and workflows Problem-solve system-level and structural challenges	Address IT resistance, with help of internal champion Test intervention and electronic information flow among systems Fidelity monitoring plans	
**MAINTENANCE (M)**						3
	**——**	Cost-benefit measures; replication costs	**——**	RE-AIM review including sustainability indicators	**——**	

a RE-AIM: Reach, Effectiveness, Adoption, Implementation, Maintenance.

b ACMs: Asthma Care Managers.

*Weights ranging from 1–9 were assigned by coders, to illustrate relative application of RE-AIM domains during planning, based on meeting agendas and minutes: 1–4 indicates = low application, 5–6 = medium application, 7–8 = high application, and 9–10 = very high application of the framework (7)*.

Moderate framework application for effectiveness (six points), was evidenced by discussions in the early stages of planning that reviewed asthma care performance indicators and patient health data to identify an appropriate intervention. Lastly, adoption and maintenance had low framework application (four points and three points, respectively), evidenced by limited discussion of what it might take for the technological intervention to be sustained beyond the study period as a part of routine care. While adoption discussions that considered Breathewell's acceptability to the ACMs and physicians involved in direct service delivery took place with relative frequency, strategic and fiscal decision-makers were not identified or discussed during planning. For example, the importance of capturing costs, a topic that is acknowledged as highly relevant to adoption and maintenance decisions at the health systems level (12), was discussed intermittently, from the perspective of costs relevant to the design and ongoing maintenance of Breathewell, should another healthcare system want to adopt it. The team also discussed quantifying the value of reallocating ACM time toward patients at higher risk for exacerbations, and reducing time spent reviewing charts and providing outreach to asymptomatic, well-managed patients based solely on their beta-agonist refill request. It was acknowledged that communicating how Breathewell added value would be necessary for its continuation after the study was completed.

### Interviews

The results of our combined RE-AIM and CFIR analyses of interview transcripts follows, organized by strongest to weakest alignment within the setting-level RE-AIM domains of implementation, adoption, and maintenance.

#### Implementation

Team reflections confirmed moderate to strong alignment with CFIR constructs associated with the Process domain. Where possible, the Breathewell intervention was designed to align with established protocols and minimize changes to existing information flows between departments. As one person confirmed, “there was really nothing within the pharmacy department that changed because where the project was really focusing on was at the junction between the pharmacy and the asthma care managers…” Also, a team member explained that manual daily monitoring of patients was instituted to assure every patient received appropriate care, and that no patient was missed, “The data pull part is essentially automated with the exception of IT issues that come up from time to time. So that part is automated but the manual checking—that takes a few minutes every morning.”

Team members agreed that involvement of stakeholders was necessary to promote their buy-in and the fit of the intervention within established workflows. Attention was paid to *engaging opinion leaders*, “[the] asthma doc helped us get any other additional signoff we [needed]…”

*Formally appointing key stakeholders* as members of the implementation planning team early on, was emphasized by several people as critical, “…it meant that the nurse, who was one of the asthma care managers, met every time we met, and she became the go-between between the [ACMs] and our team, and very much a part of our team.”

On the other hand, *engaging champions* was limited to those stakeholders who were formally appointed as members of the planning team (i.e., asthma care physician, clinical pharmacy specialist, and ACM). For example, one person noted that narrow awareness of the study presented a threat to fidelity when a group inadvertently made changes within the EHR that impacted intervention programming, stating that “… no one would have thought that changing a couple of words in a template that they (pharmacists) were using would impact this thing over here that we were doing in the research space.” In response the team instituted daily monitoring to identify unexpected problems, but acknowledged this was not a sustainable solution, “we monitor things daily … to make sure that if something does happen [that] we didn't realize was going to happen we catch it.” Another explained that settling for a monitoring vs. a programming solution was partly due to the time and budget constraints of research, “figuring out how to not have it be a research person who does so much oversight… it's a real catch-22 of how do you decide when and where to put that effort?”

Team activities and decisions that especially supported implementation fidelity, i.e., taking time for *planning* and *executing* the intervention according to protocol were evident:

…from my perspective there have been very, very few bumps along the road of big things that needed to be changed …that those things are being identified in the planning process and not waiting until they get to implementation.

Also, a clear focus of the team when designing the intervention was the need for alignment with the organization's *culture* that emphasizes patient safety and service quality, a construct associated with the Inner Setting domain:

the perspective of trying to take a more population-based approach to something, to be more all-encompassing, to make sure everyone's getting consistent care to make sure that we're reaching everyone, that it's as timely and innovative and as cost effective as it can be… I think speaks to the culture of …[the] organization. So I see it aligning really well…

CFIR constructs associated with Intervention Characteristics greatly influenced planning, with moderate to strong emphasis. Designing a technology-based intervention that required interface with patient devices, healthcare data systems, and providers was acknowledged to be highly *complex*:

So there were potential barriers –on how to extract data from the – several databases and how to integrate them and put them together, and how to fuel that or feed that over so the [intervention] would actually run and work, and how to engage patients.…

This complexity, in turn, constrained *adaptation* and *trialability*. One person said, “I think adaptations during the intervention would've been kind of difficult because it was already ‘this is how it's going to be at the beginning'.”

When asked about piloting components (i.e., *trialability*), this individual indicated that conducting a pilot was not feasible given the complex programming involved, “we did a lot …to make it work for that patient population to test it to make sure everything was working but there wasn't an actual pilot where we had like a 100 people start.”

#### Adoption

As described above, there was a strong focus on two Inner Setting domains relevant to adoption: consistency of the intervention with organizational *culture* and assuring that Breathewell was *compatible* with technology-enabled communication tools and systems already in use. One person said, “[we] looked at the goals of innovation, of good care, of using technology to our advantage…” Another commented that “we were already doing outreaches as asthma care managers. So it was part of what we were already doing.”

On the other hand, two Inner Setting CFIR constructs that would suggest a “pull” toward adopting the intervention, i.e., that it was driven by a *tension for change*, and that it was a strategic *priority*, were not supported by team reflections. As one person stated, “people weren't asking for the intervention necessarily.”

The absence of a clear pull was further confirmed when the team was asked about whether the decision to implement Breathewell was driven by research, operations, or organizational leaders, and the amount of *leadership engagement* in implementation planning. Team members agreed that the intervention was primarily based on their identification of an opportunity for improving efficiency of care, “I guess in all honestly we have to say this is our product. The [research institute] is driving the tweak that we're looking at here…”

Intervention Characteristics also were relevant to the potential post-trial adoptability of the intervention, given team-member belief that the intervention would provide a *relative advantage* to usual care by streamlining service delivery and reducing burden on ACMs. However, team members also acknowledged that it would be up to them to “push” the intervention to leadership by demonstrating that it provided a competitive advantage. “it was designed to make a difference on system effectiveness. …partially up to us to help others understand what niche we're filling.”

#### Maintenance

From a Process perspective, despite the team's care in designing the intervention so that it would be compatible with existing infrastructure and align with cultural values that the “right patients receive the right care at the right time,” there were a few “work arounds” necessitated by constraints to fully integrating the intervention into existing systems. *Planning* for post-trial modification and ongoing maintenance of Breathewell was weak. Members of the team acknowledged the intervention would need investment by the organization to fully integrate it into existing systems but had not yet evaluated the cost. One person stated that “the way it's currently structured, it's not that portable from a technical standpoint and that's probably the biggest concern I have in terms of translating it into – sustaining it in usual care.”

From an Inner Setting perspective, as described earlier, providers and staff directly involved in asthma care management were engaged throughout planning, however, higher level organizational leadership were not. Uncertainty was expressed as to which individuals or level of approvals would be needed to continue Breathewell as usual care because there was no prior commitment from operations leadership to allocate *available resources* to sustaining Breathewell after the trial.

From an Intervention Characteristics perspective, given that Breathewell is a technology-based intervention designed to improve efficiency, the team expressed the potential to promote post-trial maintenance by demonstrating its alignment with organizational priorities of optimizing efficiency without sacrificing quality:

If we can demonstrate it's cost-effective to usual care, we might be able to still have it translated, but if it turns out that there's not really any cost implications, I think in the short-term, …[there won't be interest] in doing it.

**Figure 2:**
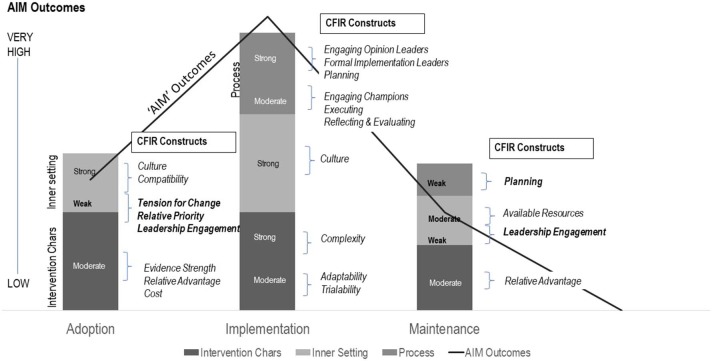
Adoption, implementation, and maintenance outcomes, explained by weak, moderate, or strong alignment with CFIR constructs. CFIR constructs were grouped within “AIM” domains, based on theory and consensus, and were assigned points to indicate their relative emphasis during planning (weak = 1; moderate = 2; strong = 3). An average “score” was then calculated for the CFIR domains of *Intervention Characteristics (Chars), Inner Setting*, and *Process* to create the stacked columns in the figure. The AIM Outcomes line graph was generated based on methods recommended by Glasgow et al. (7) for weighting relative application of RE-AIM domains by scoring them as follows: 1–4 = low application, 5–6 = medium application, 7–8 = high application, and 9–10 = very high application of the framework.

Figure 2 summarizes the relative strength of alignment between team responses to the interview questions and the sub-set of CFIR constructs deemed relevant to the setting-level RE-AIM domains of Adoption, Implementation and Maintenance.

## Discussion

“It's kind of like – you go this direction, you run into a wall, you back up, you go that direction, you run into a wall. You just keep going until you find the path.”

In this qualitative evaluation of planning for the implementation of an effective intervention of technology enhanced asthma care management, we found that formally appointing key stakeholders as planning team members, addressing workflow and system complexity, and assuring compatibility with organizational culture were key factors in promoting very high implementation fidelity. We also found that weak alignment of planning activities with Inner Setting CFIR constructs that promote leadership receptivity to interventions, such as identifying a tension for change, aligning the intervention with relative priorities, and engaging leadership in planning, likely limited post-trial adoption and maintenance of the intervention. Furthermore, by excluding CFIR constructs associated with the Outside Setting and Individual Characteristics domains, our analyses of planning overlooked potential facilitators of adoption and maintenance that may have further informed planning activities, such as competitive pressure on the organization, as well as potential barriers such as the knowledge and beliefs about the value of the intervention to the organization.

This is the first study known to us that comprehensively evaluated the planning activities and team reflections using RE-AIM and CFIR frameworks. Combining frameworks judiciously enhanced our ability to develop testable, theory-informed implementation strategies (27). Applying RE-AIM to the objective and prospectively documented meeting agendas and minutes, we observed that throughout planning the team was focused on identifying problems and solutions to ensure maximal reach of the target population, minimize disruption to existing workflows while assuring the intervention delivered patient services effectively, and with high implementation fidelity. Discussions of systems-level barriers related to the complexities of integrating a technology-based intervention into multiple electronic communication systems demonstrated the team's concerns about threats to maintaining the intervention beyond the study. However, missing from the agendas and minutes were discussions about who would ultimately need to approve and allocate organizational resources to maintain Breathewell. The result was an intervention with relatively high reach (i.e., 1080 patients (84.5%) of those potentially overusing a beta agonist, were reached for EHR assessment); and high fidelity (i.e., the Breathewell intervention was completed as designed with few exceptions). The intervention also effectively improved the efficiency of care delivery, as 41% of too-frequent asthma reliever inhaler requests were resolved by the IVR intervention (i.e., did not require ACM outreach) (24). Yet despite success in Reach, Effectiveness (i.e., improved efficiency), and Implementation, the team agreed there was low likelihood that Breathewell would be adopted and maintained. While partial use of RE-AIM (i.e., use of select domains) has been supported by its authors in recent reviews of the framework (6, 12), attention to all five RE-AIM domains during planning is necessary for implementation success. Discussions of maintenance at the onset and throughout implementation planning is particularly important for multi-year projects, to identify any unanticipated changes in the outer and/or inner setting domains that might impact organizational priorities.

When combining frameworks, it is important to ensure they can yield complementary information and avoid overly complex, conflicting or redundant sets of constructs (27). Recommendations and tools for selecting the most appropriate frameworks for different purposes are under development (28, 29) and in the future can potentially help implementation scientists select the most relevant frameworks for their specific project needs. At minimum, justification for why a framework or combination of frameworks were selected should be provided (30).

In this project, using CFIR constructs to guide team reflection illuminated the presence or absence of motivational and structural factors that, if attended to, could have improved the likelihood of adoption and maintenance. Present were characteristics of the intervention itself that were carefully aligned with organizational *culture* and assured its *compatibility* with existing infrastructure. Absent were several indicators related to *institutional climate* and *readiness*, which, if present, could potentially increase motivation or “pull” to adopt the intervention. The absent factors included lack of a known *tension for change, relative priority*, or a pre-existing agreement from leadership to commit *available resources* to fully integrate and maintain Breathewell if it was effective. The team acknowledged that, in hindsight, the lack of *leadership engagement* or *champions* outside of the implementation team and asthma care managers, (e.g., IT, pharmacy, and healthplan and asthma leadership), may have limited its full integration into organizational systems and workflows.

It was illuminating to reflect on our planning processes through a CFIR lens. However, unlike RE-AIM, where consideration of all five domains can improve implementation and dissemination outcomes across diverse interventions (31), the relevance of the CFIR domains and constructs used to explain why implementation succeeded or failed may vary by intervention and setting (32). For example, constructs associated with behavior change, such as *self-efficacy* and *individual stage of change* (33, 34) are accepted as important to human-delivered interventions but are likely less necessary for successful implementation of an automated intervention. On the other hand, post-trial adoption and maintenance of an internally developed intervention can still require demonstration of its relative advantage from a patient, competitor or regulator perspective (17). Thus, while CFIR is designed to be flexible, all five CFIR domains should be reviewed during planning to proactively identify which sets of constructs may influence full integration of the intervention into usual care, as well as receptivity or “pull” to adopt and maintain the intervention post-trial, and any constructs that are not relevant given the project.

The practice change literature recommends that organizations take time for pre-implementation planning to assure that the intervention fits within existing systems, structures, and workflows (35, 36), and can be delivered with high fidelity (2). We found that our strong focus on several key determinants of implementation success: *engagement* of key stakeholders to understand their workflow challenges; knowledge of the organization's structural *complexity; compatibility* with its complex systems; and understanding of its *culture* that prioritized patient experiences and quality of care resulted in a technology-based intervention that was executed with high fidelity. However, planning to maximize fit and fidelity was insufficient to assure post-trial adoption and maintenance, given the absence of several determinants that are associated with “pull” in the diffusion literature (3, 37). The absence of these pull factors signals a need to use targeted dissemination or “push” strategies to elicit a “pull” to adopt and maintain the intervention (3, 4). Thus, our Breathewell implementation team could possibly use “push” strategies to create “pull” by promoting its alignment with the organization's *culture*, its *compatibility* with existing systems and services, and evidence of its *relative advantage* over the status quo. A key lesson is that while proactive attention to RE-AIM and CFIR factors throughout planning is ideal, using these frameworks to guide reflection at any time during implementation could help implementation teams increase pull, by (1) communicating how the intervention will specifically fulfill organizational leaders, stakeholders and patient wants and needs; and (2) specifying what investments are necessary to assure there is organizational capacity to sustain it. A summary of our key lesson learned can be found in Table 4.

**Table 4:** Key lessons for implementation planners.

Lesson 1	Time spent in planning for implementation, that involves decision-makers and stakeholders as members of the planning team, is critical to implementation success
Lesson 2	Use of D&I frameworks both prospectively, to assess potential threats to implementation and to evaluate process and outcomes, will guide planning for implementation success
Lesson 3	No one D&I framework tells the whole story, so understanding their strengths and limits, and justifying your selection is important
Lesson 4	When using RE-AIM, all five domains enhance planning and should be monitored to assure implementation success
Lesson 5	When using CFIR, all five domains should be reviewed to identify presence or absence of relevant pull, push and infrastructure variables that can inform implementation strategies
Lesson 6	CFIR's Outer Setting domain and constructs identify relevant “pull” variables including industry trends, competitive pressure, leadership wants, and consumer demands
Lesson 7	Identifying and enlisting internal champions at all levels of the organization, who broadly promote and reinforce the value of the intervention, can facilitate implementation success

### Recommendations to Implementation Planning Teams to Improve the Odds of Implementation Success

When asked whether specific challenges were anticipated during planning, an implementation team member responded,

I've learned that there's not much rationale to sitting and trying to figure out what's going to go wrong, per se, because it'll never be that…you have to plan a process for how you're going to make a decision when something does go wrong or when you run into a barrier, but not what that specific one is.

The extreme variation in external and internal contexts, structures, and types of interventions may limit the generalizability of our specific findings about factors whose presence or absence likely influenced our adoption, implementation and maintenance outcomes. Also, factors such as the tension for change and organizational priorities may shift over the course of a multi-year study, given the dynamic context in which healthcare operates. We therefore recommend that implementation teams take the time to identify a set of relevant system- and intervention-specific determinants of adoption, implementation and maintenance, tailor their implementation strategies, and build in a process to periodically reflect and re-evaluate factors and strategies for continued relevance. Doing so will create an ongoing method for identifying and resolving problems as they occur. For specific strategies to increase RE-AIM Adoption, Maintenance, and Implementation success (see Table 5).

**Table 5:** Examples of implementation strategies recommended to address CFIR constructs and improve RE-AIM outcomes.

**Strategies to increase adoption and maintenance**	
Why should the organization invest resources in this intervention?	
**CFIR constructs**	**Implementation strategies**
Tension for change Relative priority	Engage leaders at proposal and funding stages; assess needs; identify/confirm relevant pull factors, current priorities and challenges; Increase demand for the intervention by selecting performance objectives and metrics that include at least one relevant pull factor; Develop a presentation and/or report that specifically ties the intervention to the performance objectives; clearly explains what “problem” the intervention solves; and how it supports priorities; Encourage leaders to champion or mandate the change by communicating its relative advantage and allocating resources
Track	Perceived value of, and satisfaction with, the intervention
Leadership engagement	Identify whose buy-in for implementing the intervention will be needed; Assess their understanding of the problem, and their receptivity to the proposed intervention; Increase demand for the intervention by reinforcing goal and priority alignment; Include leaders in all stages of the research including formative discussions and dissemination of findings
Track	Leadership use of process and fidelity data; reporting of feedback and findings in meetings and distribution of reports
Available resources	Identify the level of approvals that will be needed to allocate resources to modify and maintain the intervention; Determine what information (e.g., cost-benefit) they will require to commit to sustaining the change; Communicate cost-benefit data to all stakeholders
Track	Costs, cost reduction ideas, alternative funding ideas, solutions implemented
Reflecting and evaluating	Anticipate that specific preferences, needs, or demands may change given the amount of time that often elapses between proposal, funding, and study completion; Continue to engage (or re-engage) leaders throughout the study; Continue to review implementation protocols, share feedback; Disseminate progress or new evidence throughout the study, to elicit or maintain pull
Track	Changes that may impact priorities and threaten sustainability; integration of intervention into existing operations including onboarding, performance expectations, documentation, quality reports
**Strategies to increase implementation fit and fidelity**	
How do we design the intervention so that it could become a part of routine care?	
Complexity	Include internal systems experts and users in the design team; Avoid "work arounds” or have a plan (e.g., blueprint) for fully integrating the intervention into existing workflows and systems; Conduct rapid cycle tests, adapt/ refine with expert and user input
Track	Representativeness of implementation planning team; assigned roles; and extent of participation
Compatibility	Promote adaptability of intervention; Design intervention protocols to fit with existing roles and systems; Draft written protocols that can be piloted; Conduct rapid cycle tests of protocols, adapt and refine with user input; Revise written protocols to reflect user input
Track	Development and/or adaptation of written protocols, training, implementation guides
Culture	Include internal stakeholders who can identify the organizational values and norms that must be preserved by the intervention; Review workflows, training and resources for consistency with organization's values and norms; Develop and test monitoring protocols; Design and test a standard report that can be used to identify problems and address them iteratively
Track	Fidelity to established protocols, including reach and unanticipated positive or negative consequences of the intervention
OTHER	Assess for other CFIR constructs that may be relevant to implementing the proposed intervention at the System-level (e.g., networks and communication; incentives and rewards); Provider and staff-levels (e.g., knowledge and beliefs, self-efficacy); Intervention-type (e.g., adaptability, trialability)

*Strategies adapted and incorporated tips and recommendations from CFIR-ERIC Implementation Strategy Matching Tool (20) and RE-AIM key questions and tips for improving AIM performance (8)*.

## Limitations

While our use of the two frameworks in combination enabled us to not only evaluate, the *who, what, where, when* and *how* (RE-AIM) but to also explain *why* (CFIR) implementation may have succeeded or failed, particularly with regard to the presence or absence of pull factors (e.g., tension for change or peer pressure), other frameworks, such as PRISM, include contextual variables useful to adapting interventions to improve their fit and feasibility (38). Also, outcome-specific frameworks such as the Program Sustainability Assessment Tool (39), which focuses on setting-level maintenance, can be used to define the sets of conditions that need to be present or absent to sustain practice change (10, 40).

We also made some theory-informed decisions in an attempt to identify which CFIR constructs most likely influenced specific RE-AIM domains. We discovered, however, that while some constructs were well-understood as important determinants to success within a specific domain (e.g., *tension for change* for Adoption; commitment of *available resources* for Maintenance) (41); others may be relevant to performance on multiple RE-AIM domains (e.g., *leadership engagement* for Adoption and Maintenance, and *culture* for Adoption and Implementation) (1). Formally measuring the CFIR constructs and modeling them quantitatively may be useful to determine the extent specific constructs, moderated or mediated the individual RE-AIM domain outcomes (42). On the other hand, since several of the CFIR constructs overlap (e.g., leaders, stakeholders, or champions may be the same or different people, depending on their role in implementation), what or who to measure would need to be defined for the specific setting and intervention (43). It may be the case that it is not practical to measure organizations on a wide range of hypothesized determinants, and impractical to generalize which CFIR factors are determinants of which RE-AIM domains.

Our study reveals several areas for future research. First, complementary application of RE-AIM and CFIR to other implementation studies is needed to confirm the utility of using CFIR constructs to explain and improve performance on RE-AIM domains. Second, applying analytic methods, such as qualitative comparative analysis (10), to compare sets of factors or conditions that are sufficient or necessary to implementation success, may help to inform appropriate implementation strategies. Third, using measures to quantitively model the pathways in which CFIR factors moderate (pull) or mediate (push) RE-AIM results may lead to an integrated conceptual model that will improve their complementary use. Last, experimentally testing implementation strategies designed to promote conditions favorable to implementation success, such as those recommended in Table 5, will contribute to improving the effectiveness of implementation planning.

## Conclusions

Our study addresses an important gap in implementation science—illustrating how complementary application of evaluation (RE-AIM) and explanatory (CFIR) frameworks can identify the presence or absence of variables necessary for implementation success. This approach demonstrated that attention to factors important to maximizing the fit of an intervention within a healthcare system, and monitoring patient receipt of the most appropriate services, yielded an intervention with high reach and implementation fidelity, but low likelihood of post-trial adoption or maintenance. We identified modifiable CFIR constructs that could improve receptivity to adopt and maintain evidence-based interventions. We recommend early assessment and attention to these constructs to inform tailoring of implementation strategies to maximize implementation success.

## Data Availability Statement

The datasets generated for this study are available on request to the corresponding author.

## Ethics Statement

The studies involving human participants were reviewed and approved by Institutional Review Board at Kaiser Permanente Colorado. Written informed consent for participation was not required for this study in accordance with the national legislation and the institutional requirements.

## Author Contributions

DK and JS developed the design plan, selection of frameworks, analytic approach, development of interview questions, and a priori codes. JS and CA assisted with compiling and coding of historical documents and conducted the implementation team interviews. DK and JS coded and analyzed intervention transcripts, which were validated by BB, MR, DR, NW, and CA. All authors contributed content, provided feedback on tables and figures, reviewed the manuscript, and contributed to the concept and design of the study.

### Conflict of Interest

The authors declare that the research was conducted in the absence of any commercial or financial relationships that could be construed as a potential conflict of interest.
